# Novel strain properties distinguishing sporadic prion diseases sharing prion protein genotype and prion type

**DOI:** 10.1038/srep38280

**Published:** 2017-01-16

**Authors:** Laura Cracco, Silvio Notari, Ignazio Cali, Man-Sun Sy, Shu G. Chen, Mark L. Cohen, Bernardino Ghetti, Brian S. Appleby, Wen-Quan Zou, Byron Caughey, Jiri G. Safar, Pierluigi Gambetti

**Affiliations:** 1 Department of Pathology, Case Western Reserve University, Cleveland, Ohio, United States of America; 2 National Prion Disease Pathology Surveillance Center, Case Western Reserve University, Cleveland, Ohio, United States of America; 3 Department of Pathology and Laboratory Medicine, Division of Neuropathology, Indiana University, Indianapolis, Indiana, United States of America; 4 Department of Neurology, Case Western Reserve University, Cleveland, Ohio, United States of America; 5 Department of Psychiatry, Case Western Reserve University, Cleveland, Ohio, United States of America; 6 Laboratory of Persistent Viral Diseases, NIH/NIAID Rocky Mountain Laboratories, Hamilton, Montana, United States of America

## Abstract

In most human sporadic prion diseases the phenotype is consistently associated with specific pairings of the genotype at codon 129 of the prion protein gene and conformational properties of the scrapie PrP (PrP^Sc^) grossly identified types 1 and 2. This association suggests that the 129 genotype favours the selection of a distinct strain that in turn determines the phenotype. However, this mechanism cannot play a role in the phenotype determination of sporadic fatal insomnia (sFI) and a subtype of sporadic Creutzfeldt-Jakob disease (sCJD) identified as sCJDMM2, which share 129 MM genotype and PrP^Sc^ type 2 but are associated with quite distinct phenotypes. Our detailed comparative study of the PrP^Sc^ conformers has revealed major differences between the two diseases, which preferentially involve the PrP^Sc^ component that is sensitive to digestion with proteases (senPrP^Sc^) and to a lesser extent the resistant component (resPrP^Sc^). We conclude that these variations are consistent with two distinct strains in sFI and sCJDMM2, and that the rarer sFI is the result of a variant strain selection pathway that might be favoured by a different brain site of initial PrP^Sc^ formation in the two diseases.

It is now well established that the basic mechanism of prion diseases relies on the conversion of the normal or cellular prion protein (PrP^C^) to the abnormal and infectious conformer commonly identified scrapie PrP (PrP^Sc^)^1^. A challenging aspect of this conversion mechanism is that it leads to the formation of heterogeneous PrP^Sc^ species commonly identified as strains^2^. Historically, strains have been defined as prion species that upon transmission to receptive hosts are associated with different disease phenotypes as determined by distinct incubation periods, histological lesions and PrP deposits with respect to type and topography^3^. Subsequently, strains were correlated to PrP^Sc^ species exhibiting distinct physicochemical properties, such as electrophoretic mobility, which were attributable to different conformations linked to any primary to quaternary structure variation^4^,^5^,^6^,^7^,^8^.

Human prion diseases are characterized by a marked phenotypic heterogeneity that has hampered their recognition and understanding^9^. Although this heterogeneity is due in part to the unique presence of three etiologies – sporadic, inherited and acquired by infection – the sporadic group alone includes at least seven phenotypes^2^,^10^. We proposed a classification of sporadic human prion diseases based on the pairing of the patient’s genotype at codon 129 of the PrP gene (the site of a common methionine (M)/valine (V) polymorphism) with the type, 1 or 2, of PrP^Sc^ (as determined by electrophoretic mobility of the protease resistant PrP^Sc^ core or resPrP^Sc^)^11^,^12^. Individual 129 genotype - PrP^Sc^ type pairs consistently correlate and identify most sporadic prion disease phenotypes^2^,^10^,^12^. For example, sporadic Creutzfeldt-Jakob disease (sCJD) affecting 129MM patients and associated with PrP^Sc^ type 1 (identified as sCJDMM1) exhibits a clinical and pathological phenotype that significantly differs from the phenotypes associated with sCJDVV1 or sCJDMM2^12^. However, this principle does not apply to all sporadic prion diseases.

Sporadic fatal insomnia (sFI, also identified as sCJDMM2T, for thalamic form), and sCJDMM2 (also identified as sCJDMM2C, for cortical form) are both associated with the 129MM genotype and PrP^Sc^ conformers of type 2, which display a similar resPrP^Sc^ profile in both diseases upon standard electrophoretic analysis. However, in spite of sharing molecular features, sFI and sCJDMM2 are associated with strikingly different clinical and histopathological phenotypes as well as prevalences (Supplementary Table S1). In sFI the major lesions are concentrated to the thalamus and consist of severe loss of neurons; in contrast, sCJDMM2 is characterized by severe spongiform degeneration made of distinctive large vacuoles that preferentially affect the cerebral cortex^10^,^12^. Bioassay studies indicate that the two diseases are associated with distinct prion strains^13^. Detailed studies of resPrP^Sc^ have shown consistent differences which, however, involve very minor fragments, raising questions as of whether these minor variations can bring about such drastic phenotypic differences^14^,^15^. Furthermore, systematic comparative studies of the physicochemical characteristics of the PrP^Sc^ associated with the two diseases are missing.

We searched for variations of PrP^Sc^ properties that could explain the striking phenotypic heterogeneity of sFI and sCJDMM2. We found major variations in the protease-sensitive component of PrP^Sc^, while novel although less striking differences also emerged in resPrP^Sc^.

## Results

### Glycoform ratios and electrophoretic patterns of totPrP^Sc^ and resPrP^Sc^

**Figure 1:**
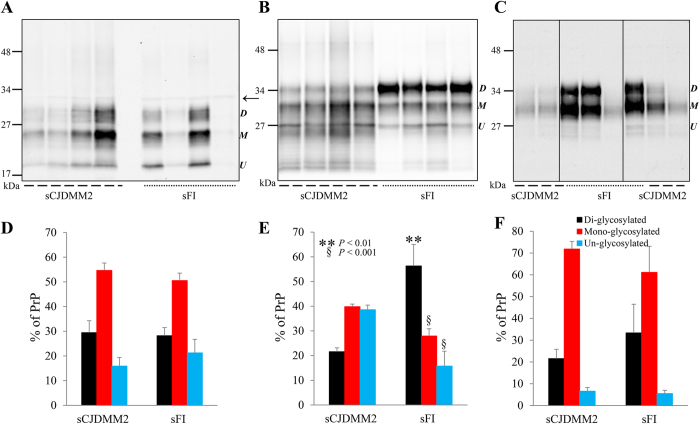
Western blot profiles and glycoform ratios of PK-treated and untreated PrP^Sc^, as well as presumed PrP^C^, from sCJDMM2 and sFI. BH from sCJDMM2 and sFI were precipitated with NaPTA, and the totPrP^Sc^-containing pellets were either PK-treated (PK 10 U/ml) generating resPrP^Sc^ (**A**) or left untreated (totPrP^Sc^) (**B**). The percent distribution of the glycoforms of resPrP^Sc^ and totPrP^Sc^ harvested from NaPTA precipitates are shown in the bar graphs (**D**,**E**). (**A**,**B**,**D**,**E**) Unlike resPrP^Sc^, totPrP^Sc^ shows WB profiles that differ in the two diseases because of a significant over-representation of the di-glycosylated form in sFI. In (**A**), a non-specific band of ~32 kDa is also observed (arrow). Following NaPTA precipitation, the supernatants were precipitated in methanol and resuspended in sample buffer. (**C**,**F**) Supernatants from sCJDMM2 and sFI, run on the same gel but not in adjacent lanes, show similar ~27–37 kDa WB profiles and percent distribution of the glycoforms of “PrP^C^” (*P* > 0.05, Student’s *t*-test). In sFI, the “PrP^C^” glycoform ratio is significantly different from that of totPrP^Sc^ (**B**,**E**), indicating that the totPrP^Sc^ ratio is not due to contamination with “PrP^C^”. (Labels on the right side of each WB indicate the three PrP glycoforms: *D*: Di-glycosylated; *M*: Mono-glycosylated; *U*: Un-glycosylated. Tissue samples from four cases of sCJDMM2 and sFI, respectively, and the mAb to PrP, 3F4, have been used in all figures, unless otherwise indicated).

Comparative examination on Western blots (WB) of resPrP^Sc^ obtained from frontal cortex and enriched by sodium phosphotungstate (NaPTA) precipitation confirmed the lack of qualitative differences between sFI and sCJDMM2 electrophoretic profiles with regard to mobility and ratios of the three resPrP^Sc^ glycoforms (Fig. 1A,D).

However, differences were demonstrated when the proteinase K (PK) treatment of enriched samples was omitted, allowing for the examination of total PrP^Sc^ (totPrP^Sc^) [i.e. resPrP^Sc^ + PK-sensitive PrP^Sc^ (senPrP^Sc^) = totPrP^Sc^]. Total PrP^Sc^ revealed a significant variation in the ratios of the di-glycosylated, mono-glycosylated and un-glycosylated conformers that respectively showed the 56:28:16 ratios in sFI and 21:40:39 in sCJDMM2 (Fig. 1B,E). The possibility that the different glycoform ratios of totPrP^Sc^ reflected significant contamination of the NaPTA precipitate with PrP^C^ was considered to be very unlikely in view of the different glycoform ratios exhibited by the PrP (presumably PrP^C^) recovered in the NaPTA supernatant (Fig. 1C,F). Similar results were obtained from the medial thalamus, the cerebral region most affected in sFI (Supplementary Fig. S1).

**Figure 2:**
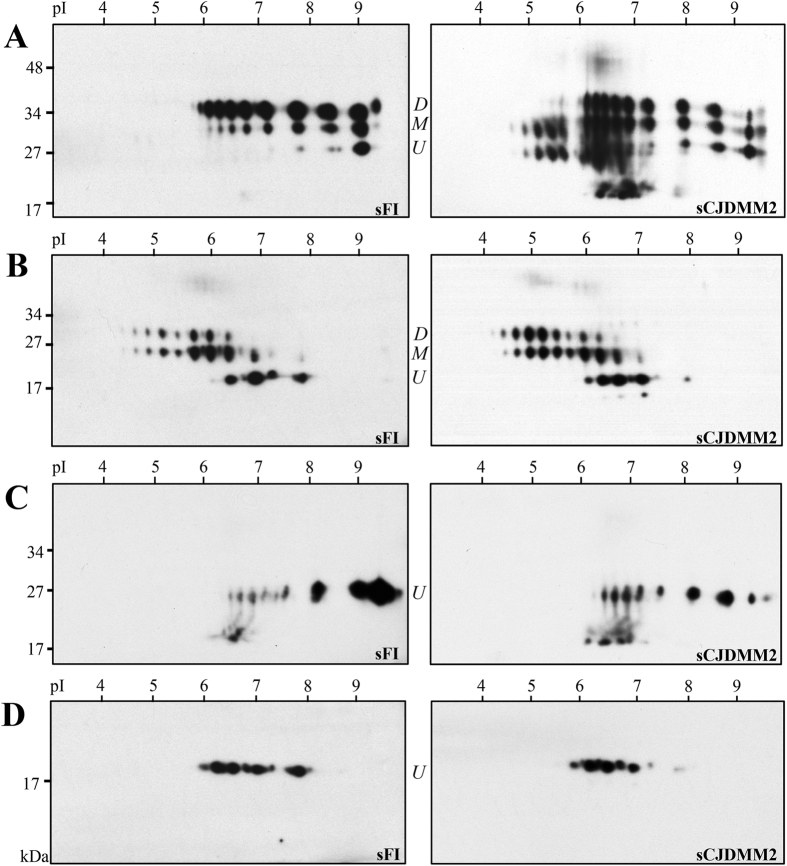
Two-dimensional WB of PrP^Sc^ isoforms from sFI and sCJDMM2. TotPrP^Sc^ (**A**), resPrP^Sc^ (**B**), PNGase F-deglycosylated totPrP^Sc^ (**C**) and deglycosylated resPrP^Sc^ (**D**) were processed for 2-D WB following BH precipitation with NaPTA in sFI and sCJDMM2 as indicated. (**A**) TotPrP^Sc^ shows a much more complex spot pattern in sCJDMM2 than in sFI, the pattern of which resembles that of PrP^C^ 17. The patterns become much simpler and similar in the two diseases following treatment with PK (**B**), PNGase F (**C**) or both treatments combined (**D**). (Labels on the side of each WB indicate the three PrP glycoforms: *D*: Di-glycosylated; *M*: Mono-glycosylated; *U*: Un-glycosylated; PK 10 U/ml).

Further characterization of totPrP^Sc^ and resPrP^Sc^ on two-dimensional (2-D) WB demonstrated marked differences in totPrP^Sc^ harvested from sFI and sCJDMM2 with regard to the number and gel mobility of the electrophoretic spots (Fig. 2A). With regard to totPrP^Sc^, the 2-D polyacrylamide gel electrophoresis (PAGE) pattern was relatively simple in sFI, but quite complex in sCJDMM2, as it included PrP^Sc^ isoforms with wide ranges of relative molecular weights and isoelectric points. This disparity was especially prominent in the 6.0–7.0 isoelectric points (pI) range. In this region of the sCJDMM2 preparations, the spots corresponding to the three glycoforms were blurred by a smear comprised in the 40–20 kiloDalton (kDa) range. Furthermore, a prominent cluster of spots could be seen in the 19 kDa region while a weak but large, blurred spot occupied the ~50 kDa high molecular weight (h.m.w.) area. Besides seemingly confirming the ratios of the three totPrP^Sc^ glycoforms observed in one-dimensional (1-D) WB, differences in pI were uncovered in the two diseases, due to the spanning over a wider range of pI for glycoforms of sCJDMM2 totPrP^Sc^. Following PK treatment, the sCJDMM2 electrophoretic pattern became much simpler, matching that of sFI (Fig. 2B). In both diseases, resPrP^Sc^ spots conspicuously shifted toward the acidic region compared to those of totPrP^Sc^. Patterns were even further simplified and variations between sCJDMM2 and sFI further reduced after deglycosylation of totPrP^Sc^ and resPrP^Sc^ with PNGase F, although minor differences persisted (Fig. 2C,D). Altogether, these 2-D WB findings confirm that the major heterogeneity between sFI and sCJDMM2 PrP^Sc^, especially with respect to the ratios and pI of the glycoforms, is related to the totPrP^Sc^ species, pointing to senPrP^Sc^ as the component accountable for the diversity.

### Characterization of full-length totPrP^Sc^

**Figure 3:**
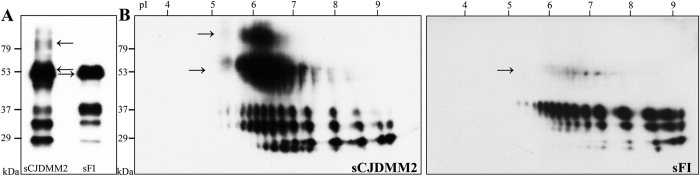
One- and 2-D WB of full-length totPrP^Sc^ immunoprecipitated from sCJDMM2 and sFI. Immunoprecipitation was carried out on NaPTA pellets with the mAb 8B4 to PrP N-terminus. (**A**) The 1-D WB shows h.m.w. bands (arrows) especially prominent in sCJDMM2 preparations, in addition to the three typical PrP^Sc^ glycoforms; (**B**) In 2-D WB, spots corresponding to the h.m.w. are dramatically represented in sCJDMM2 and barely detectable in sFI (arrows). Note that in these full-length totPrP^Sc^ preparations, low molecular weight fragments are lacking but the glycoform ratios reproduce those of the whole totPrP^Sc^ (Fig. 2A).

To further investigate the complexity of the totPrP^Sc^ in sCJDMM2, we immunoprecipitated the full-length component of totPrP^Sc^ from NaPTA preparations using a monoclonal antibody (mAb) to the PrP N-terminal region (Fig. 3). The 1-D WB of the immunoprecipitate showed that the three full-length PrP^Sc^ glycoforms associated with sFI and sCJDMM2 reproduced the ratios previously observed on preparations of whole totPrP^Sc^ (Figs 3A and 1B,E). In addition, the eluate demonstrated the presence of two large h.m.w. bands of ~53 kDa and ~90 kDa in sCJDMM2 while a single, less well represented ~53 kDa band was detectable in sFI (Fig. 3A). On 2-D WB, the two ~53 kDa and ~90 kDa h.m.w. components were very prominent in sCJDMM2 preparations, where they occupied a large area of the blot at approximately pI 6.5, while the ~53 kDa component alone was barely noticeable in sFI (Fig. 3B). Presence, relative quantity and PrP^Sc^ nature of the ~53 kDa h.m.w. were confirmed following immunoprecipitation (IP) or in NaPTA preparations (i.e. omitting IP), using different elution conditions to exclude co-elution of the capturing mAb, and by probing with polyclonal antibodies to the C- and N-termini as well as with the conformational mAb OCD4 (Supplementary Figs S2 and S4B)^16^. The ~53 kDa and ~90 kDa h.m.w. components were found to be glycosylated and partially PK-sensitive; the ~53 kDa form was estimated to be 3–4 times better represented in sCJDMM2 than in sFI (Supplementary Fig. S2F–I).

When the 2-D WB patterns of whole and full-length totPrP^Sc^ from sCJDMM2 were compared, the pattern of the full-length totPrP^Sc^ isoform (presence of the ~53 kDa and ~90 kDa spots notwithstanding) appeared much simpler than that of the whole totPrP^Sc^ (which also comprised the truncated forms), especially in the pI region where the two h.m.w. components were located (Figs 2A and 3B). In contrast to those of sCJDMM2, totPrP^Sc^ 2-D patterns obtained from sFI were comparable, regardless of whether they exclusively represented the full-length totPrP^Sc^ isoform or the whole totPrP^Sc^ comprising full-length and truncated forms (Figs 2A and 3B). In conclusion, the 2-D study of full-length totPrP^Sc^ (i) confirms the presence in sCJDMM2 totPrP^Sc^ of significant amounts of ~53 kDa and ~90 kDa h.m.w. species, which appear to contain full-length totPrP^Sc^; (ii) indicates that the PrP^Sc^ fragments that complicate the WB 2-D pattern of totPrP^Sc^ in sCJDMM2 originate from the h.m.w. components (Figs 2A and 3B); and (iii) confirms the variations of glycoform ratios and pI in sCJDMM2 and sFI, which are especially noticeable in the full-length un-glycosylated component (Fig. 3B).

### Sedimentation properties of prion aggregates

**Figure 4:**
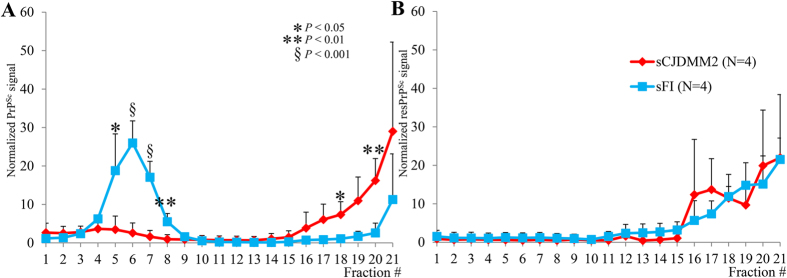
Sedimentation equilibrium of totPrP^Sc^ and resPrP^Sc^. TotPrP^Sc^ from sCJDMM2 and sFI were centrifuged at high speed for 19 hours in a 10–60% sucrose gradient. Identical volumes were collected from each fraction and processed for WB. Sedimentation profiles of totPrP^Sc^ (**A**) and resPrP^Sc^ **(B**). Note the distinct peak of totPrP^Sc^ low-density aggregates in sFI and the prevalence of high-density aggregates in totPrP^Sc^ from sCJDMM2 (**A**). In contrast, only high-density aggregates are demonstrated in resPrP^Sc^ (**B**). (PK 10 U/ml).

PrP^Sc^ variations distinguishing sCJDMM2 and sFI were further searched with sedimentation equilibrium (SE) and sedimentation velocity (SV) centrifugations, which are commonly used to explore density and size of the PrP^Sc^ aggregates, respectively. Following SE, totPrP^Sc^ sedimentation profiles clearly differed between the two diseases: in sCJDMM2, over 70% of totPrP^Sc^ aggregates populated the sucrose high-density region of the gradient (bottom fractions), while in sFI, over 80% of the totPrP^Sc^ was recovered in a distinct peak located in a lower density region (fractions 3–8) (Fig. 4A). Remarkably, unlike those of sCJDMM2, sFI glycoform ratios varied significantly along the gradient (Supplementary Fig. S3). This variation resulted in a significant difference between the glycoform ratios recovered in the low- and high-density fractions in sFI, and in a difference in ratios between the two diseases in the low- but not in the high-density fractions (Supplementary Fig. S3B).

Following PK digestion of individual SE fractions, the signal of fractions 3–8 pertaining to sFI was no longer detectable, while the only sizable quantity of resPrP^Sc^ aggregates populated the high-density fractions in both diseases (Fig. 4B). Thus, PK treatment removed all significant differences between the two gradient profiles although minor differences remained in the glycoform ratios (Fig. 4B and Supplementary Fig. S5). In contrast, when PK digestion was carried out on the whole totPrP^Sc^ preparations before SE (rather than on the individual SE fractions), two distinct although overlapping aggregate populations were observed that involved fractions of lower density (fractions 8–18) in sFI than in sCJDMM2 (12–21) (Supplementary Fig. S6).

**Figure 5:**
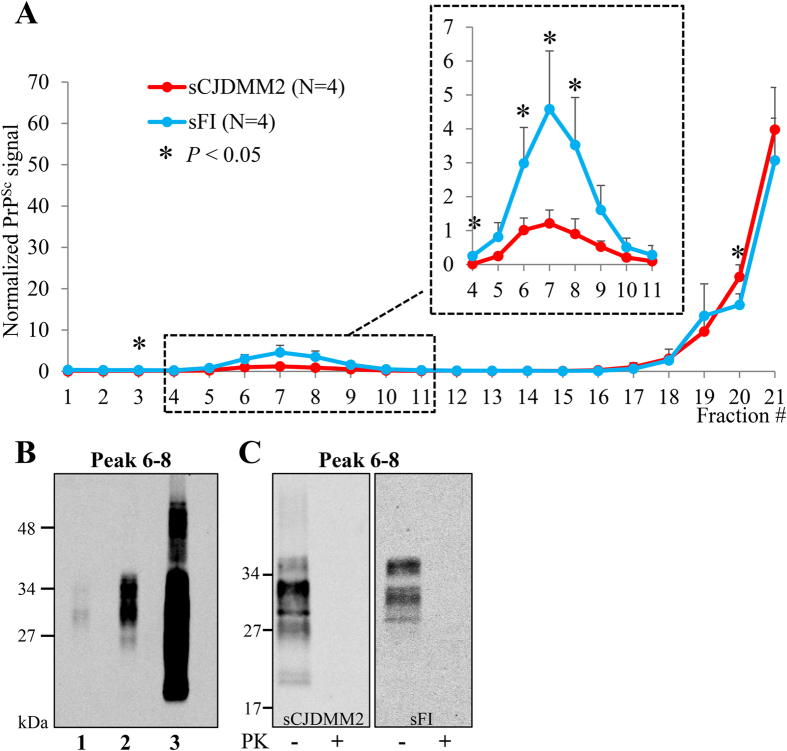
Sedimentation equilibrium of totPrP^Sc^ and insoluble PrP used as control obtained under stringent conditions. Total PrP^Sc^ was harvested under stringent conditions from identical BH volumes of sCJDMM2 and sFI, while PrP, likely iPrP, was similarly obtained from cases free of neurological diseases used as controls. Both preparations were centrifuged at high speed for 19 hours in a 10–60% sucrose gradient. (**A**) Sedimentation profiles of totPrP^Sc^ in sCJDMM2 and sFI showing a slight prevalence of low-density aggregates in sFI and high-density aggregates in sCJDMM2. (**B**) WB of fractions 6–8 combined, from controls, sFI and sCJDMM2 (lanes 1, 2, 3, respectively), harvested from the same brain equivalents, excludes the possibility that the low-density peaks in sFI and sCJDMM2 (lanes 2, 3) are populated by iPrP only^17^ (lane 1). (**C**) Fractions 6–8, normalized to the same total protein concentration and exposed to limited proteolysis (PK 0.031 U/ml), are PK-sensitive in both diseases and display the same difference in glycoform representation as in non-stSE conditions (Supplementary Fig. S3).

Experiments on control cases indicated that totPrP^Sc^ recovered in P2 from sFI and sCJDMM2 and used for SE might have been contaminated up to 16% by PrP^C^, insoluble PrP (iPrP)^17^, or both (Cracco *et al*., unpublished data). Therefore, SE was repeated using totPrP^Sc^ P2 preparations generated under stringent conditions (indicated as stSE, see Supplementary Materials and Methods) and harvested from sCJDMM2 and sFI as well as from brains free of neurological diseases. Although following stSE the majority of sCJDMM2 and sFI totPrP^Sc^ aggregates populated the high-density region of the gradient, smaller but still significant differences in their respective distributions were also detected in the low-density area of the gradient: in sCJDMM2, only 4% of totPrP^Sc^ was recovered in fractions 5–10, whereas in sFI this component exceeded 14% (*P* < 0.05); a significant difference was also found in the high-density regions of the gradient (fractions 17–21, *P* < 0.05) (Fig. 5A). WB of stSE fractions 6–8 combined, which were harvested from brain equivalents of negative controls (presumably comprising PrP^C^, iPrP or both) as well as from sFI and sCJDMM2, indicated that the contribution of PrP^C^ or iPrP (lane 1) was insignificant, excluding the possibility that the low-density peaks in sFI and sCJDMM2 (lanes 2, 3) were due to contamination (Fig. 5B). Furthermore, major SE characteristics of low- and high-density fractions (6–8 and 17–21), like PK sensitivity and variations of glycoform ratios observed along the gradient, were reproduced in stSE, indicating that totPrP^Sc^ exhibited similar qualitative features in SE and stSE gradients (Fig. 5C and Supplementary Fig. S3, and Cracco *et al*., unpublished data). It should also be noted that the stSE procedure caused a significant loss of totPrP^Sc^ to the S2 fractions, which impacted preferentially sFI and may explain the quantitative variations in aggregate distribution between the two SE methods (Cracco *et al*., unpublished data).

The possibility that in SE the low-density peak observed in sFI reflected contamination of totPrP^Sc^ with PrP^C^ or iPrP (expected to populate similar gradient fractions and to be PK-sensitive) seemed also unlikely in view of the results of the IP experiments with mAb OCD4, the conformational antibody reacting with misfolded PrP species but not with PrP^C^ 16. Immunoprecipitation of PrP from the 4–8 and 17–21 SE fractions obtained from sFI and sCJDMM2 yielded significant quantities of PrP displaying glycoform ratios similar to those of the totPrP^Sc^ directly collected from the same SE and stSE fractions (Supplementary Figs S3 and S4). In contrast, OCD4 captured relatively negligible amounts of PrP, possibly iPrP, in S1 and S2 fractions generated from a negative case (Supplementary Fig. S4)^17^. Of note, h.m.w. components populated both low- and high-density fractions in sCJDMM2 but only the high-density fractions in sFI (Supplementary Fig. S4B).

**Figure 6:**
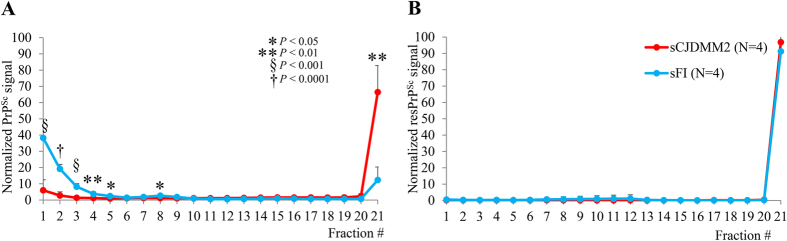
Sedimentation velocity of totPrP^Sc^ and resPrP^Sc^. TotPrP^Sc^ from sCJDMM2 and sFI cases were subjected to 1 hour of high-speed centrifugation in a 5–15% sucrose gradient. Individual fractions were processed for WB untreated (**A**) or following PK treatment (10 U/ml) (**B**). (**A**) TotPrP^Sc^ aggregates preferentially populate low-density fractions in sFI and the highest density fraction in sCJDMM2. (**B**) No significant difference is found between the profiles generated by resPrP^Sc^ in the two diseases.

Following SV centrifugation (1 hr, in 5–15% sucrose gradient), the sedimentation profiles were again different in the two diseases (Fig. 6). In sFI, virtually all totPrP^Sc^ aggregates were confined to the low-density fractions (fractions 1–5), while in sCJDMM2 only approximately 10% of totPrP^Sc^ distributed in that region, while nearly 70% was recovered in the highest density fractions of the gradient (Fig. 6A). Treatment with PK equalized the profiles since aggregates were exclusively recovered in the highest density fraction in both diseases, further adding to the notion that most PrP^Sc^ disparity resides in the senPrP^Sc^ fraction (Fig. 6B).

### Solubility properties of totPrP^Sc^ and resPrP^Sc^

**Figure 7:**
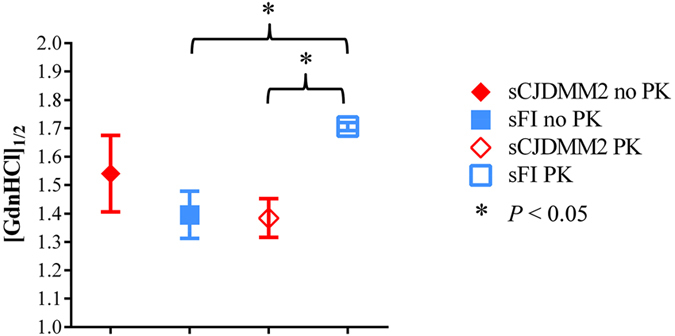
Conformational solubility and stability assay of totPrP^Sc^ and resPrP^Sc^. [GdnHCl]_1/2_ represents amounts (in molar values) required to solubilize half of the substrate and is used as an indication of conformational stability. The mean [GdnHCl]_1/2_ values for totPrP^Sc^ and resPrP^Sc^ are 1.54 M and 1.38 M in sCJDMM2, and 1.40 M and 1.71 M in sFI. Although the stability values of totPrP^Sc^ are comparable in the two diseases, the stability of resPrP^Sc^ in sFI is significantly higher than that of the corresponding totPrP^Sc^ as well as that of resPrP^Sc^ associated with sCJDMM2.

Finally, both totPrP^Sc^ and resPrP^Sc^ associated with sCJDMM2 and sFI were further characterized by the conformational solubility and stability assay (CSSA) that assesses conformational stability based on the rate of PrP^Sc^ solubilisation at increasing concentrations of guanidine hydrochloride (GdnHCl)^18^,^19^. In sCJDMM2, totPrP^Sc^ and resPrP^Sc^ showed comparable solubility values; in sFI, while the totPrP^Sc^ solubility roughly matched that of sCJDMM2, resPrP^Sc^ was significantly less soluble than totPrP^Sc^ as well as the resPrP^Sc^ of sCJDMM2 (Fig. 7). Therefore, conformational stability is another feature differentiating resPrP^Sc^ in sFI and sCJDMM2.

## Discussion

We have uncovered significant variations affecting totPrP^Sc^ and, to a lesser extent, resPrP^Sc^ conformers that distinguish sFI and sCJDMM2. The variations appear to affect primarily the senPrP^Sc^ component of totPrP^Sc^, as they drastically diminish when the totPrP^Sc^ preparations are digested with PK to reveal resPrP^Sc^. The variations of totPrP^Sc^ involve a variety of properties, including (i) ratio and isoelectric point of the glycoforms, (ii) presence of two ~53 kDa and ~90 kDa h.m.w. prominent components and a plethora of low m.w. PrP^Sc^ fragments as well as (iii) existence of an aggregate population displaying distinct density and, possibly, size (Supplementary Table S2). The variations affecting primarily resPrP^Sc^ comprise (i) distinct densities of aggregates, which are detected when PK treatment is carried out before (rather than after) SE fractionation, and (ii) conformational stability (Supplementary Table S2). Furthermore, lower PK resistance of totPrP^Sc^ in sFI compared to that of sCJDMM2 has been reported by Saverioni *et al*.^20^. The finding that totPrP^Sc^ diversity in the two diseases appears to be mostly determined by the senPrP^Sc^ component may explain the enduring lack of recognition of significant PrP^Sc^ heterogeneity in these two diseases.

The observation that in sFI (but not in sCJDMM2) the glycoform ratio of totPrP^Sc^ is dominated by the di-glycosylated form and differs from that of resPrP^Sc^ argues that, in sFI, senPrP^Sc^ and resPrP^Sc^ preferentially target different PrP^C^ glycoforms for conversion^21^,^22^,^23^,^24^,^25^. It has recently been proposed that the sialic acid moiety of the PrP^C^ glycans impairs conversion to PrP^Sc^ 24. Therefore, the dominance of the di-glycosylated (thus highly sialylated) isoform in sFI totPrP^Sc^, presumably involving the senPrP^Sc^ component, might reflect a lower sialylation (or other variations) of PrP^C^ glycans available for conversion to senPrP^Sc^ in sFI, allowing for the conversion of even the PrP^C^ di-glycosylated isoform. Alternatively, PrP^C^ conversion to senPrP^Sc^ might be less affected by sugar sialylation in sFI, or senPrP^Sc^ and resPrP^Sc^ glycoforms might have different turnovers. Regardless of the mechanism, the difference in glycoform ratios is significant considering the rising evidence that glycan representation, structure or both can affect strain characteristics^21^,^25^,^26^.

The high representation of the ~53 kDa and ~90 kDa h.m.w., and of the low molecular weight (l.m.w.) components in totPrP^Sc^ from sCJDMM2 only, strikingly demonstrated in 2-D WB of totPrP^Sc^, is puzzling. Our preliminary characterization indicates that the two components share several physicochemical features, suggesting that l.m.w. fragments derive from the h.m.w. component. Immunoprecipitation experiments and probing with a number of PrP antibodies indicate that the ~53 kDa h.m.w. component contains glycosylated, full-length PrP and may represent PrP dimers (the ~90 kDa would consist of trimers) or aggregates that are covalently linked. Most of the h.m.w. and l.m.w. components appear to be insoluble in detergents and, at least in part, PK-sensitive, suggesting that they mostly comprise senPrP^Sc^. High m.w. PrP components, similar to the ~53 kDa we observed in sCJDMM2, have been reported previously^18^,^20^,^27^,^28^,^29^. Priola and colleagues^28^ originally described a 60 kDa PrP, covalently-linked dimer in neuroblastoma cells expressing hamster PrP and in scrapie-infected hamster brains. A PrP^Sc^ dimer has also been observed in a variety of conditions including infected and non-infected transgenic mice expressing mutant PrP, detergent-insoluble preparations from sCJDMM2 brain homogenates and from bank voles inoculated with the aforementioned brain homogenate, but not bank vole normal brains^18^,^20^,^29^. Remarkably, the dimer presence in totPrP^Sc^ of sCJDMM2-inoculated bank voles implies that the dimer is transmissible and therefore is an intrinsic characteristic of the sCJDMM2 strain.

Furthermore, the 2-D experiments also confirmed the distinct glycoform ratios observed in 1-D WB and uncovered the difference in pI of the glycoforms associated with totPrP^Sc^ and resPrP^Sc^ in sCJDMM2 and sFI. Somewhat similar patterns were observed in a previous 2-D WB comparative study of full-length totPrP^Sc^ in sFI and sCJDMM2^14^,^30^. These studies, however, did not report the presence of h.m.w. electrophoretic spots perhaps due to methodological differences^14^,^30^.

The disparity of senPrP^Sc^ aggregates that we observed between sFI and sCJDMM2, following fractionation by long and short sucrose density centrifugations, is also noteworthy since it relates to the current notion that aggregate profiles are good identifiers of distinct strains^31^. The long (19 hrs.) SE centrifugations, thought to primarily assess density (mass/volume), showed that most totPrP^Sc^ co-distributes with low-density aggregates in sFI and with aggregates of high density in sCJDMM2. However, the detergent insoluble fractions used in this SE protocol included up to 16% contamination with non-PrP^Sc^ (PrP^C^ and insoluble PrP). A more stringent SE protocol still demonstrated a significant difference between the distribution of sCJDMM2 and sFI low-density aggregates, although this protocol led to a loss of totPrP^Sc^ that predominantly affected senPrP^Sc^ in sFI. Furthermore, the dominant presence of senPrP^Sc^ in the sFI low-density fractions was further supported by the distinct glycoform ratios and by the immunoreactivity of the PrP recovered in these fractions using a conformational antibody that recognizes totPrP^Sc^ and resPrP^Sc^ but not PrP^C^. Although further study is needed, an appealing possibility is that the sFI low-density aggregates match the protease-sensitive small oligomers recently described and are associated with detergent-insoluble cholesterol-rich lipids originating from the PrP^Sc^ association with membrane rafts^32^. Furthermore, association with lipids is thought to play a role in maintaining PrP^Sc^ conformation and controlling strain features^33^,^34^,^35^,^36^. Notably, the sFI (but not the sCJDMM2) glycoform ratios changed significantly along the gradient, regardless of the type of SE used, implying that in sFI the low- and high-density aggregates are composed of monomers that differ with respect to glycosylation. A further distinctive feature is that the h.m.w. components appeared to populate both low- and high-density fractions in sCJDMM2 but only the high-density fractions in sFI. Although caution has to be exercised because of the different representation of the h.m.w. components in the two diseases, this finding suggests that, in addition to representation, h.m.w. components differ also with respect to their participation in aggregate formation in sFI and sCJDMM2. Protease treatment of the individual fractions removed all differences between sFI and sCJDMM2, indicating that all low-density fractions 1–10 harboured senPrP^Sc^ while resPrP^Sc^ populated mostly the high-density fractions 11–21, in amounts directly related to the density of the fractions. However, when PK digestion of totPrP^Sc^ was carried out before SE fractionation (rather than on the individual fractions), two distinct, though overlapping, aggregate populations were observed in sFI and sCJDMM2, which accounted for over 80% of the entire resPrP^Sc^ aggregates in both conditions. The different resPrP^Sc^ aggregate distribution under this PK treatment condition likely denotes partial re-aggregation of resPrP^Sc^ that results in distinct aggregate populations in the two diseases. It also underlines the importance of the timing of PK treatment in SE^31^.

Sedimentation velocity, which is supposed to separate aggregates by size, showed, like SE, that the great majority of totPrP^Sc^ aggregates populated the light fractions in sFI, while in sCJDMM2 the majority was recovered in the fractions of highest density. This sedimentation procedure generated an almost symmetrical bimodal profile due to the nearly complete separation of light fractions (1–5 in decreasing representation) from the 20–21 heavy fractions, suggesting that in our SV conditions aggregates appear similar in size in both diseases but are differently represented, with the smaller aggregates predominating in sFI. Following protease treatment of the individual fractions, aggregates were almost exclusively recovered in the densest fractions. However, in view of its minimalist bimodal profile, our SV procedure might have failed to fully separate aggregates in sFI and sCJDMM2, making the interpretation of the sedimentation profile challenging. Nonetheless, SV experiments further confirm the dissimilarity of totPrP^Sc^ aggregates in sFI and sCJDMM2.

Sedimentation velocity studies of PrP^Sc^ have recently been carried out in sCJD subtypes by Saverioni *et al*.^20^. The SV profiles of sCJDMM2 and sFI in this study profoundly differ from ours especially due to the underrepresentation of the low-density component in sFI. However, Saverioni *et al*.^20^ carried out SV fractionation on PrP^Sc^ preparations (P3) which were purified under stringent conditions by performing two rounds of over 2-hour high speed centrifugations, and sonication. Nonetheless, a significant difference in aggregate distribution was observed between sFI and sCJDMM2^20^.

The major finding obtained with the conformational solubility and stability assay was that while the stabilities of totPrP^Sc^ were similar in both diseases, the resPrP^Sc^ stability was significantly higher in sFI not only when compared with that of the corresponding totPrP^Sc^ but also with respect to the stability of resPrP^Sc^ in sCJDMM2. A possible mechanism of the enhanced stability of sFI PrP^Sc^ following PK treatment is re-aggregation of resPrP^Sc^ promoted by PK treatment, which is different in the two diseases and results in increased stability of resPrP^Sc^ in sFI but not in sCJDMM2. This mechanism is consistent with the drastically different SE aggregate profiles engendered when PK digestion is performed on the totPrP^Sc^ before SE (a condition similar to that of the CSSA procedure) as opposed to the PK digestion of the SE-generated individual fractions. Alternatively, assuming that the stability of totPrP^Sc^ represents the average of the stabilities of both senPrP^Sc^ and resPrP^Sc^ components, the high stability of the resPrP^Sc^ even without PK treatment might compensate for the very low stability of senPrP^Sc^. Should this be the case, stabilities of both senPrP^Sc^ and resPrP^Sc^ associated with sFI would differ from those of sCJDMM2. Regardless of these interpretations, the CSSA test uncovers an additional unexpected disparity of resPrP^Sc^ in sCJDMM2 and sFI. Stability data comparable to ours have been reported for sCJDMM2 PrP^Sc^ in previous studies^18^,^37^, along with the finding that, in sCJDMM2, senPrP^Sc^ is more stable than resPrP^Sc^ 32.

A bioassay study by Moda *et al*.^13^ showing that sCJDMM2 and sFI have quite distinct transmission properties also supports the diversity of the strains associated with these two diseases according to the classic definition of strain^3^. On the other hand, the notion that distinct strains generally associate with distinct phenotypes and vice versa is supported by the finding that following bioassay or direct characterization no major differences were observed in totPrP^Sc^ and resPrP^Sc^ from sCJDMM1 and sCJDMV1, two subtypes of sCJD that, despite the different genotype at codon 129, show no significant phenotypic disparity^12^,^38^. These considerations raise at least two key questions: (i) Which of the PrP^Sc^ variations in sFI and sCJDMM2 do encrypt the basic phenotypic differences of these two diseases? (ii) How can these variations be compatible with the current concepts of strain formation and evolution? Although future studies will provide more precise answers to the first question, the different representations of major components of totPrP^Sc^ like the individual glycoforms and the dimers, the differing stabilities of resPrP^Sc^ and the different aggregate profiles of both totPrP^Sc^ and resPrP^Sc^ point to the presence of fundamental differences in the two diseases involving PrP^Sc^ tertiary and quaternary structures, or the modalities of the PrP^C^ to PrP^Sc^ conversion, both of which can likely specify distinct phenotypes^22^,^35^,^39^,^40^.

Concerning the compatibility of our findings with current prion strain notions, it is widely accepted that prion strains initially form as a spectrum of conformers, which are then prioritized through a process of Darwinian selection, with the dominant component triggering the disease and imparting its phenotypic characteristics^41^,^42^,^43^,^44^. Low compatibility of all strains with PrP^C^ may require a conformational change of the PrP initially converted or lead to the targeting of selected PrP^C^ conformers to allow propagation^45^,^46^. Caution must certainly be used in applying these notions (largely acquired from animal and cell experimentation) to sporadic human prion diseases in which the initial PrP^Sc^ is formed *de novo*, in the absence of an exogenous strain serving as template, and seemingly in circumscribed brain regions^47^. Nevertheless, the coexistence of significant amounts of PrP^Sc^ types 1 and 2 in about 40% of 129MM cases, combined with the finding that the disease phenotype reflects the ratio of the two types, is consistent with the presence of a selection process resulting in the co-existence of a dominant strain with sub-strains also in human sporadic prion diseases^37^,^48^,^49^,^50^,^51^. Other remarkable features of human sporadic prion diseases are that the variety of phenotypes and the major PrP^Sc^ species with which they are associated, regularly recur with very similar features and consistent prevalences despite the apparent lack of template. Furthermore, as mentioned above, the PrP genotype, determined by the methionine/valine polymorphism at codon 129, is a strong determinant of the prion strain. For example, in sCJD approximately 86% of the affected subjects who are methionine homozygous (sCJDMM) carry exclusively or predominantly PrP^Sc^ type 1, while the remaining 14% have PrP^Sc^ type 2 as the exclusive or prevalent type. Accordingly, the sCJDMM1 phenotype accounts for about 86% of all cases of sCJDMM while sCJDMM2 and sFI phenotypes account for up to 11.9% and 1.8%, respectively^10^,^37^, (unpublished data). According to the spectrum hypothesis, it is tempting to speculate that all three PrP^Sc^ strains (i.e. type 1MM; type 2MM and type 2MM sFI variant) are initially present in sCJDMM and sFI patients, but PrP^Sc^ type 1 is preferentially selected while the sFI strain very rarely is.

The preferential brain region where the initial PrP^C^ to PrP^Sc^ conversion takes place might also play a role in strain selection and PrP^Sc^ properties, especially when the influence of the 129 polymorphism is expected to have no discriminating role as in sCJDMM2 and sFI^52^. Indirect evidence points to the thalamus as the locale of the earliest lesions in sFI, while the severe involvement of the cerebral cortex as for histopathology and PrP^Sc^ accumulation argues that the brunt of the conversions process in sCJDMM2 might take place in the cortex^53^,^54^. To deepen the current understanding of the early events in the pathogenesis and phenotypic determination in sporadic prion diseases it would be important to gain insight into how the brain locale (i.e. the biological environment) of the initial PrP^C^ to PrP^Sc^ conversions is chosen and the role it plays in strain diversity.

## Materials and Methods

See Supplementary Materials and Methods for a list of reagents and antibodies used, and for description of the following: molecular genetics, prevalence, clinical and histopathological evaluations, methanol and methanol-chloroform precipitations, deglycosylation by PNGase F, IP with 8B4, antibody co-elution, IP with OCD4, 1- and 2-D electrophoresis and immunoblot, preparation of detergent insoluble fraction with or without stringent conditions, sedimentation equilibrium and velocity.

### Brain tissues and Ethics statement

Frozen brain tissues were obtained at autopsy from the National Prion Disease Pathology Surveillance Center (NPDPSC); they included frontal cortex from controls not affected by any neurological disease (N = 3), and frontal cortex and thalamus from confirmed cases of sCJDMM2 (N = 4) and sFI (N = 4). All procedures were performed under protocols approved by the Institutional Review Board at Case Western Reserve University. Written informed consent for research was obtained from all patients or legal guardians according to the Declaration of Helsinki. All patients’ data and samples were coded and handled in accordance with NIH guidelines to protect patients’ identities.

### Preparation of brain homogenates

Brain homogenates (BH) (10% w/v) were prepared in cold lysis buffer (LB) 100 pH 8.0 (100 mM NaCl, 0.5% Nonidet P-40, 0.5% sodium deoxycholate, 10 mM EDTA, 100 mM Tris-HCl, pH 8.0)^55^. Homogenization was carried out with 5 × 75 sec. cycles with high-energy cell disrupter Mini-Beadbeater-16 (BioSpec), allowing 1 min interval at 4 °C between each cycle. Solubilization was optimized by freezing the samples between cycles 3 and 4.

### Sodium phosphotungstate precipitation

The original NaPTA precipitation protocol^7^ was modified to improve PrP^C^ solubilization and reduce PrP^C^ contamination in the precipitate. Homogenates were cleared by 5 min 500 × g centrifugation at 4 °C. 10% cleared homogenates, mixed 1:1 with a solution 16% sarkosyl NL in 2X D-PBS without CaCl_2_ and MgCl_2_ at pH 7.4, were well vortexed. For further details refer to Supplementary Materials and Methods.

### Proteinase K digestion

Samples in LB 100 pH 8.0 were incubated at 37 °C for 1 hour with PK (58 U/mg specific activity, 1 U/ml equal to 17.2 μg/ml PK] that was adequate to efficiently digest both PrP^C^ and senPrP^Sc^ (data not shown). The reaction was stopped by the addition of 3 mM PMSF. Unless otherwise indicated, PK digestion was performed in samples harvested from the same brain equivalents.

### Determination of total protein concentration

Pierce BCA Protein Assay Kit was used following the manufacturer’s instructions with minor modifications.

### Conformational stability and solubility assay

CSSA was performed as originally described^18^ with minor modifications^19^. For further details refer to Supplementary Materials and Methods.

### Statistical analysis

Unpaired, two-tailed Student’s *t*-test was used, after determination of equal or unequal variance between the samples. The level of statistical significance was indicated: *P < 0.05; **P < 0.01; ^§^*P* < 0.001; ^†^*P* < 0.0001.

## Additional Information

**How to cite this article**: Cracco, L. *et al*. Novel strain properties distinguishing sporadic prion diseases sharing prion protein genotype and prion type. *Sci. Rep.* **7**, 38280; doi: 10.1038/srep38280 (2017).

**Publisher's note:** Springer Nature remains neutral with regard to jurisdictional claims in published maps and institutional affiliations.

## Supplementary Material

Supplementary Information
