# Supplementary material for: Novel strain properties distinguishing sporadic prion diseases sharing prion protein genotype and prion type
Source: Sci Rep. 2017 Jan 16;7:38280. doi: 10.1038/srep38280 (PMC5238384; doi:10.1038/srep38280)

SUPPLEMENTARY INFORMATION

Novel strain properties distinguishing sporadic prion diseases sharing prion protein genotype and prion type

Laura Cracco, Silvio Notari, Ignazio Cali, Man-Sun Sy, Shu G. Chen, Mark L. Cohen, Bernardino Ghetti, Brian S. Appleby, Wenquan Zou, Byron Caughey, Jiri G. Safar, Pierluigi Gambetti*

* Address correspondence to: Pierluigi Gambetti, pxg13@case.edu

SUPPLEMENTARY MATERIALS AND METHODS

Reagents and antibodies

NaCl, Nonidet P-40, sodium deoxycholate, Tris-HCl, phosphate buffered saline (PBS), Dulbecco's PBS (D-PBS), N-Lauroylsarcosine sodium salt solution (sarkosyl NL), sodium phosphotungstate octadecahydrate, $MgCl_2$ –hexahydrate, phenylmethanesulfonyl fluoride (PMSF), aprotinin, leupeptin, proteinase K, sucrose, thiourea, cOmplete™ Ultra Protease Inhibitor Cocktail Tablets, Kodak Biomax MR and XAR films were purchased from Sigma-Aldrich (St. Louis, MO, USA); sodium dodecyl sulfate (SDS), β–mercaptoethanol, Tween 20, urea, 3-[(3-cholamidopropyl)dimethylammonio]-1-propanesulfonate (CHAPS), tributylphosphine (TBP), iodoacetamide, ReadyPrep 2-D Cleanup Kit, dithiothreitol (DTT), Triton X-100, Bio-Lyte 3/10 Ampholyte, 15% Criterion Tris-HCl polyacrylamide precast gels, Ready-Strip IPG Strips pH 3-10, 8–16% Criterion Tris-HCl polyacrylamide precast gels, bromophenol blue, Non-fat dry milk, Tris-buffered saline (TBS) from Bio-Rad Laboratories (Hercules, CA, USA). Odyssey Blocking Buffer from LI-COR Biosciences (Lincoln, NE, USA); methanol, chloroform, glycerol, GdnHCl solution, Pierce™ BCA Protein Assay Kit, Dynabeads Antibody Coupling Kit and Dynabeads M-280 Tosylactivated from Thermo Fisher Scientific Inc. (Waltham, MA, USA); ethylenediaminetetraacetic acid (EDTA) from Promega (Madison, WI, USA); glycerol-free PNGase F from New England Biolabs Inc. (Ipswich, MA, USA); polyvinylidene difluoride (PVDF) membrane (Immobilon-P or Immobilon-FL) from EMD Millipore (Billerica, MA, USA) and ECL and ECL plus reagents from GE Healthcare Life Sciences (Piscataway, NJ, USA). The antibodies used were: mouse mAb 3F4 (to human PrP residues 106-110)[1,2], rabbit anti-C-terminal antiserum 2301 (to human PrP residues 220-231)[3,4], mAb 8B4 (to human PrP residues 36-43)[5], rabbit anti-N-terminal antiserum anti-N (to human

PrP 23-40) and anti-DNA mAb OCD4[6]. Secondary antibodies included infrared Dye (IRDye) 800CW goat anti-mouse IgG and IRDye 680RD goat anti-rabbit IgG (LI-COR Biosciences, Lincoln, NE, USA); goat anti-mouse IgG (Fc) horseradish peroxidase (HRP) conjugate from Thermo Fisher Scientific Inc. (Waltham, MA, USA); sheep anti-mouse and donkey anti-rabbit IgG, HRP-linked whole antibodies from GE Healthcare, Life Sciences (Piscataway, NJ, USA).

Molecular genetics

DNA was extracted from the frozen brain tissue and the *PRNP* coding region analysed as previously described[7,8].

Prevalence, clinical and histopathological evaluations

All sporadic 129 MM cases diagnosed by histopathological or histopathological and WB examinations at the NPDPSC during the 2005- 2014 period were selected. Cases of VPSPr were excluded; cases of sCJDMM1-2 (we observed no sFI1-2 cases) were attributed to the sCJDMM1 or sCJDMM2 categories according to the predominant phenotype[9]. Prevalences of sFI and sCJDMM2 are expressed as percentage of these two conditions with respect to the total number of sporadic 129MM cases.

Retrospective chart review was carried out for all subjects paying particular attention to the clinical presentation of the disease.

Histology and immunohistochemistry were carried out as previously described[10]. Brain sections from frontal, temporal, parietal and occipital neocortices, hippocampus, neo-striatum, thalamus and cerebellar hemisphere were assessed for presence and severity of spongiform degeneration, neuronal loss and astrogliosis. Immunohistochemistry was performed with mAb 3F4 to determine the presence and pattern of PrP^{Sc} deposition.

Methanol precipitation

Samples were mixed 1:5 with pre-chilled methanol, vortexed and incubated at -20°C for at least 2 hours before centrifugation at 16,000 x g for 30 min at 4°C. The pellets were resuspended in buffer.

Methanol-chloroform precipitation

NaPTA pellets were incubated with 4-fold volumes of methanol-chloroform (2:1) solution and centrifuged at 16,000 x g for 30 min at 4°C; they were then resuspended in buffer.

Deglycosylation by PNGase F

PNGase F was used as recommended by the manufacturer. Briefly, NaPTA or methanol pellets were resuspended in 1X Glycoprotein Denaturing Buffer and incubated at 100°C for 10 min. After addition of Nonidet P-40 and G7 Reaction Buffer, PNGase F was added; the mixture was incubated overnight at 37°C. PNGase F was then heat-inactivated at 75°C for 10 min.

Immunoprecipitation with 8B4

According to manufacturer's instructions, Dynabeads M-270 Epoxy (5 mg, ~3.35×10^8 beads) were conjugated to 100 µg of purified mAb 8B4 using the Dynabeads Antibody Coupling Kit reaching a final concentration of 10 mg 8B4-coupled beads/ml. Prior to IP, cleared homogenates from sCJDMM2 and sFI cases were subjected to NaPTA and methanol-chloroform precipitations; the final pellets were resuspended in 15 µl of 8 M GdnHCl solution, sonicated and heated at 80°C for 5 min. These samples were then diluted to 100 µl with LB 100 pH 8.0 containing protease inhibitors (1 mM PMSF and 5µg/ml aprotinin and leupeptin), and were incubated with ~2.35×10^7 8B4-coupled beads and a solution containing 3% Tween 20, 3% Nonidet P-40 in 1X PBS pH 7.4, to reach a reaction volume of 1 ml. The tubes were incubated with tilting and rotation overnight at 4°C, and placed on a magnet, allowing the beads to pellet completely; they were then washed nine times with a solution containing 2% Tween 20, 2%

Nonidet P-40 in 1X PBS pH 7.4. Then, a final wash with 1X PBS pH 7.4 was performed. In cases of PK treatment, the beads were resuspended in 30 µl of LB 100 pH 8.0 and incubated with PK (5 U/ml) for 1 hour at 37˚C constantly mixing; the reaction was blocked with 3 mM PMSF prior to the addition of 2X sample buffer and elution. Two alternative protocols were used for antigen elution of PK-untreated samples: i) incubation of the beads in 1X sample buffer (1.05% SDS, 13.2% glycerol, 0.005% bromophenol blue, 2.5% β –mercaptoethanol, 32.9 mM Tris–HCl, pH 6.8) and heating at 96°C for 6 min (PK-treated samples were directly boiled after addition of 2X sample buffer); ii) incubation of the beads in urea elution buffer (8 M urea, 2% CHAPS, 20 mM Tris pH 8.0) for 1 hour at room temperature with constant mixing. In both cases the beads were then separated from the eluate using a magnet. The eluates in 1X sample buffer were boiled at 100°C for 10 min whereas the samples in urea elution buffer were first reduced with 5 mM TBP, then alkylated with 20 mM iodoacetamide and then subjected to the ReadyPrep 2-D Cleanup Kit which reduces detergents, salts and lipids. The pellets were resuspended in rehydration buffer (7 M urea, 2 M thiourea, 1% DTT, 1% CHAPS, 1% Triton X-100, 0.2 % Bio-Lyte 3/10 ampholyte) for isoelectric focusing (IEF) applications, but where indicated they were resuspended in either 1X sample buffer or 1X Glycoprotein Denaturing Buffer.

Antibody co-elution

To test whether covalent bonding between 8B4 and Dynabeads M-270 Epoxy formed during coupling prevented antibody leakage during the elution, sCJDMM2 and sFI were subjected to IP-8B4; after the last wash with 1X PBS pH 7.4, the beads were separated in two tubes and eluted either in 1X sample buffer or urea elution buffer. For samples eluted in urea elution buffer, ReadyPrep 2-D Cleanup Kit was used and the resulting pellets were resuspended in 1X sample buffer and boiled at 100°C for 10 min. The samples were then subjected to 1-D electrophoresis

and immunoblot with i) mAb 3F4; ii) rabbit anti-N-terminal antiserum anti-N; iii) rabbit anti-C-terminal antiserum 2301; iv) goat anti-mouse IgG (Fc) HRP conjugate (that was also used as secondary Ab for 3F4 detection); and v) donkey anti-rabbit IgG HRP-linked whole antibody (also used as secondary Ab for anti-N and 2301 detection).

Immunoprecipitation with OCD4

IP with OCD4 was performed as described[6], with minor modifications. OCD4-coupled beads were incubated with similar amounts of PrP in each sample. Samples referred to as fractions 4-8 and 17-21 were obtained by combining the contents of the selected fractions obtained with SE experiments from N=2 cases of sCJDMM2 and sFI. S1 and S2 samples (see "Preparation of detergent insoluble fraction") were obtained from a case unaffected by neurological disease. Elution was performed by heating the beads at 95°C for 5 min in SDS sample buffer (3% SDS, 2 mM EDTA, 10% glycerol, 50 mM Tris-HCl, pH 6.8).

1-D electrophoresis and immunoblot

Proteins were separated with 15% Criterion Tris-HCl polyacrylamide precast gels and blotted into Immobilon-FL PVDF membranes for 2 hours at 60V, which were then rinsed with 1X PBS and blocked with Odyssey Blocking Buffer for 1 hour at room temperature before being probed overnight, at 4°C. The primary antibodies were diluted in Odyssey Blocking Buffer with 0.1% Tween 20 as follows: 3F4 (1:40,000 except for SE, SV and CSSA in which it was used at a dilution of 1:4,000); anti-N (1: 2,000); 2301 (1:3,000) and 8B4 (1:10,000, stock 2 mg/ml). After four washes with PBS-T (1X PBS with 0.1% Tween 20), the membranes were incubated for 1 hour with IRDye 800CW goat anti-mouse IgG (1:15,000) or IRDye 680RD goat anti-rabbit IgG (1:15,000), diluted in Odyssey Blocking Buffer with 0.1% Tween 20 and 0.01% SDS and protected from light until development. After four washes with PBS-T and a 5 min wash with 1X

PBS, membranes were developed by Odyssey Classic infrared imaging system (LI-COR Biosciences), as described by the manufacturer. Densitometric analysis was performed using the Odyssey application software V3.0 (LI-COR Biosciences). After normalization, the data were plotted and expressed as mean ± standard deviation.

For IP experiments immunoblots were developed by chemiluminescence, requiring the use of a compatible membrane (Immobilon-P PVDF), blocking and antibody incubation buffers (5% non-fat dry milk in Tris-buffered saline with 0.1% Tween 20 (TBS-T)), HRP-conjugated secondary antibodies and chemiluminescent substrate for developing (further details under 2-D electrophoresis and immunoblot).

2-D electrophoresis and immunoblot

The first dimension (isoelectric focusing) of 2-D PAGE was performed as previously described with minor modifications[11]. Briefly, 11 cm Ready-Strip IPG Strips pH 3-10 were rehydrated for 15 hours with the samples and subjected to isoelectric focusing with PROTEAN IEF Cell (Bio-Rad), for ~ 40,000 Vh. The IPG Strips were then equilibrated in SDS and reducing/alkylating agents in a two-step process by the addition of 130 mM DTT and 135 mM iodoacetamide, respectively, to the equilibration buffer (6M urea, 2% SDS, 20% glycerol, 0.375 M Tris-HCl pH 8.8), prior to running the second dimension gels. Criterion Tris-HCl polyacrylamide precast gels (8–16%) were used. Proteins were transferred into Immobilon-P PVDF membranes for 2 hours at 60 V; the membranes were then blocked with 5% non-fat dry milk in TBS-T for 1 hour at room temperature before being incubated overnight, at 4°C, with mAb 3F4 (1:40,000). After five washes with TBS-T, the membranes were incubated with goat anti-mouse IgG (Fc)-HRP conjugate (1:5,000) for 1 h at room temperature, washed again and developed by enhanced

chemiluminesce reaction using ECL and ECL plus reagents, as recommended by the manufacturer. The signal was captured on Kodak MR and XAR films.

Sodium phosphotungstate precipitation

After addition of a protease inhibitors cocktail (final concentration 1mM PMSF and 5µg/ml aprotinin and leupeptin), the 5% homogenates were incubated with a solution 10% (w/v) NaPTA/85 mM $MgCl_2$ (final concentration 0.32% NaPTA/2.72 mM $MgCl_2$) for at least 1h at 37˚C while shaking. A supernatant and a pellet were recovered by 14,000 x g centrifugation for 30 min at 20˚C. The supernatants were stored at -80˚C and the pellets were washed in 1X D-PBS pH 7.4 before their resuspension in buffer. For 2-D PAGE, pellets were precipitated by methanol-chloroform.

Preparation of detergent insoluble fraction

BH (20% w/v) in 1X Dulbecco's PBS pH 7.4 were mixed with an equal volume of 2X LB100 pH 8 (200 mM NaCl, 1% Nonidet P-40, 1% sodium deoxycholate, 20 mM EDTA, 200 mM Tris-HCl, pH 8.0), incubated at least 10 min at 4˚C and centrifuged at 1,000 x g for 10 min at 4°C. The supernatant obtained, identified as S1, was then ultracentrifuged at 100,000 x g for 1 h at 4°C in a SW55 rotor (Beckman) to generate a supernatant (S2) and pellet (P2) for each sample[12]. The P2 were resuspended in cold LB 100 pH 8.0; the S2 were stored in -80°C.

Preparation of detergent insoluble fraction under stringent conditions

Stringent conditions were used to maximize PrP^C solubility and to guarantee that in the resulting P2 the level of PrP^C contamination was less than 1% of the total PrP contained in the BH. Briefly, BH (20% w/v) in 1X TEND (65 mM NaCl, 0.5 mM EDTA, 10 mM Tris pH 8.0) were mixed with an equal volume of a solution 20% sarkosyl NL in 1X TEND containing a protease inhibitor cocktail, and incubated at 4˚C for 30 min with frequent mixing. S1, obtained by

centrifuging the samples at 18,000 rcf for 25 min at 4°C, were subsequently centrifuged at 100,000 x g for 2 h at 4°C in a SW55 rotor to generate S2 and P2 obtained under stringent conditions[13], with minor modifications. The P2 were either resuspended in LB 100 pH 8.0 or 1% sarkosyl NL TNE pH 7.2 (150 mM NaCl, 5 mM EDTA, 25 mM Tris pH 7.5).

Sedimentation equilibrium

SE was performed with the intent of separating the particles based on their individual densities[14]. P2 fractions containing comparable PrP^{Sc} amounts (determined by immunoblot with 3F4) or originating from identical volumes of BH (brain equivalents) were loaded on top of step sucrose gradients (10% to 60%), prepared in 1% sarkosyl NL TNE pH 7.2. The P2 samples [either PK-treated (PK 10 U/ml) or –untreated] were subjected to ultracentrifugation at 200,000 x g for 19 hours at 4°C in a SW55 rotor (Beckman). For each sample, 21 fractions were collected from the top of the tube, the content of which was concentrated by methanol precipitation and resuspended in LB 100 pH 8.0 by sonication in ice. SE generated using insoluble fractions prepared under stringent conditions were indicated as stringent conditions SE. The samples were then either treated with PK (as separate fractions of identical volume or as a combination of selected fractions, when indicated) or directly mixed with an equal amount of 2X sample buffer and boiled at 100°C for 10 min. Identical volumes of each fraction were then subjected to immunoblot with 3F4 and densitometry analysis. The signal in each fraction was normalized as percent of the total and plotted as mean ± SD for each group. Graph lines identified the sedimentation curves for each sample group. Fraction 21 was excluded from the glycoform ratio analysis due to smearing of bands.

Sedimentation velocity

SV was performed to assess the distribution of PrP^{Sc} aggregates in a gradient according to size[14]. The protocol used was comparable to that of SE with two major exceptions: i) time of centrifugation (1 hour instead of 19 hours); ii) type of sucrose gradient (5-15% instead of 10-60%). The choice of a different range of sucrose concentrations was based on the results obtained with SE, with the goal of having SV comparisons based primarily on particle size, thus using a gradient less dense than the particles that were attempted to be separated by size. Incubation of P2 samples in LB containing 1% sarkosyl NL prior to PrP^{Sc} sedimentation did not affect the SV profile.

Conformational stability and solubility assay

Five aliquots of P2 in LB 100 pH 8 (30µl), from the same brain equivalents (w/v), were diluted 1:1 with GdnHCl solutions to obtain final GdnHCl concentrations ranging between 0 M and 4 M. The samples were incubated at 37˚C for 1 h while mixing and then centrifuged at 22˚C for 20 min at 16,000 x g. Since insoluble PrP^{Sc} was the focus of the investigation, pellets were resuspended in 1X sample buffer and boiled for 10 min at 100˚C. In addition, and different from the original protocol, the assay was also applied to PK-treated P2 samples, to compare the solubility curves of $resPrP^{Sc}$ with those of $totPrP^{Sc}$. PK digestion (PK 5 U/ml) was performed before incubation with GdnHCl solutions[15]. Finally, all samples were subjected to immunoblotting with 3F4. After densitometry analysis using Odyssey application software V3.0 and normalization, individual dose-response best-fitted denaturation curves and $[GdnHCl]_{1/2}$ values were determined using GraphPad Prism 6 (GraphPad Software). The mean $[GdnHCl]_{1/2}$ values for each group ± standard error were determined and plotted.

References

1 Kascsak, R. J. *et al.* Mouse polyclonal and monoclonal antibody to scrapie-associated fibril proteins. *J Virol* **61**, 3688-3693 (1987).

2 Zou, W. Q. *et al.* PrP conformational transitions alter species preference of a PrP-specific antibody. *J Biol Chem* **285**, 13874-13884, doi:10.1074/jbc.M109.088831 (2010).

3 Monari, L. *et al.* Fatal familial insomnia and familial Creutzfeldt-Jakob disease: different prion proteins determined by a DNA polymorphism. *Proc Natl Acad Sci U S A* **91**, 2839-2842 (1994).

4 Chen, S. G. *et al.* Truncated forms of the human prion protein in normal brain and in prion diseases. *J Biol Chem* **270**, 19173-19180 (1995).

5 Li, R. *et al.* Identification of an epitope in the C terminus of normal prion protein whose expression is modulated by binding events in the N terminus. *J Mol Biol* **301**, 567-573, doi:10.1006/jmbi.2000.3986 (2000).

6 Zou, W. Q., Zheng, J., Gray, D. M., Gambetti, P. & Chen, S. G. Antibody to DNA detects scrapie but not normal prion protein. *Proc Natl Acad Sci U S A* **101**, 1380-1385, doi:10.1073/pnas.0307825100 (2004).

7 Parchi, P. *et al.* Molecular basis of phenotypic variability in sporadic Creutzfeldt-Jakob disease. *Ann Neurol* **39**, 767-778, doi:10.1002/ana.410390613 (1996).

8 Parchi, P. *et al.* Genetic influence on the structural variations of the abnormal prion protein. *Proc Natl Acad Sci U S A* **97**, 10168-10172 (2000).

9 Cali, I. *et al.* Co-existence of scrapie prion protein types 1 and 2 in sporadic Creutzfeldt-Jakob disease: its effect on the phenotype and prion-type characteristics. *Brain* **132**, 2643-2658, doi:10.1093/brain/awp196 (2009).

10 Pastore, M. *et al.* Creutzfeldt-Jakob disease (CJD) with a mutation at codon 148 of prion protein gene: relationship with sporadic CJD. *Am J Pathol* **167**, 1729-1738, doi:10.1016/S0002-9440(10)61254-0 (2005).

11 Yuan, J. *et al.* Insoluble aggregates and protease-resistant conformers of prion protein in uninfected human brains. *J Biol Chem* **281**, 34848-34858, doi:10.1074/jbc.M602238200 (2006).

12 Zou, W. Q. *et al.* Identification of novel proteinase K-resistant C-terminal fragments of PrP in Creutzfeldt-Jakob disease. *J Biol Chem* **278**, 40429-40436, doi:10.1074/jbc.M308550200 (2003).

13 Bolton, D. C., Bendheim, P. E., Marmorstein, A. D. & Potempska, A. Isolation and structural studies of the intact scrapie agent protein. *Arch Biochem Biophys* **258**, 579-590 (1987).

14 Rhodes, D. G., Bossio, R. E. & Laue, T. M. Determination of size, molecular weight, and presence of subunits. *Methods Enzymol* **463**, 691-723, doi:10.1016/S0076-6879(09)63039-1 (2009).

15 Cali, I. *et al.* Co-existence of PrP^{Sc} types 1 and 2 in sporadic CJD with genotype 129VV. *Prion* **6 Supplement**, 134-135 (2012).

Supplementary Table S1. Prevalence, clinical and histopathological characteristics of sFI and sCJDMM2.

Type and Case #	Gender	Age at Onset (years)	Duration (months)	Clinical Presentation	Neuropathology	Prevalence *
sFI # 1	Male	43	25	Depression, psychosis	Minimal spongiform degeneration (SD) in cerebral cortex with occasional status spongiosus. Severe loss of neurons with astroglial reaction in the medial thalamus with no SD. Focal regions of PrP immunostaining in the superficial cerebral cortex with a dot- and microplaque-like pattern	NA
sFI # 2	Female	60	12	Parkinsonism, supranuclear palsy		NA
sFI # 3	Male	36	25	Cerebellar impairment, supranuclear palsy		NA
sFI # 4	Male	45	35	Memory impairment, generalized pruritus	As # 1-3 but with moderate SD in basal ganglia and thalamus. Neuronal loss, astrogliosis and moderate SD in medial anterior thalamus. Scattered moderate PrP immunostaining in basal ganglia and brainstem with plaque-like pattern	NA
**Average sFI**	**NA**	**46 ± 10**	**24 ± 9**	**NA**	**NA**	**1.8%**
sCJDMM2 # 1	Male	65	15	Memory impairment, weight loss	Moderate to severe and widespread SD with large vacuoles involving predominantly the cerebral cortex. Moderate SD with minimal loss of neurons in the anterior thalamus. Intense and widespread coarse PrP immunostaining with peri-vacuolar and plaque-like patterns	NA
sCJDMM2 # 2	Female	72	15	Memory impairment, myoclonus, ataxia		NA
sCJDMM2 # 3	Female	57	12	Visual and memory impairment		NA
sCJDMM2 # 4	Female	37	19	Memory impairment, myoclonus, ataxia		NA
**Average sCJDMM2**	**NA**	**58 ± 15**	**15 ± 3**	**NA**	**NA**	**11.9%**

* Expressed as percentage of sFI and sCJDMM2 cases with respect to the total number of sporadic 129MM cases (see Supplementary Materials and Methods for details).

Supplementary Table S2. Variations and similarities of totPrP^{Sc} and resPrP^{Sc} between sFI and sCJDMM2.

Study	totPrP^{Sc}		resPrP^{Sc}	
	**sFI**	**sCJDMM2**	**sFI**	**sCJDMM2**
Glycoform ratios (di-:mono-:un-glycosylated PrP^{Sc})	56:28:16 *	21:40:39 *	28:51:21	29:55:16
2-D WB profiles	24-26 species *	53-65 species *	29 species	30 species
~53 kDa & ~90kDa components	± *	+++ *	NA	NA
Aggregate distribution after SE (PrP^{Sc} representation in fractions 1-10)	81% *	22% *	11%	7%
Aggregate distribution after SV (PrP^{Sc} representation in fractions 1-10)	81% *	18% *	5%	2%
Conformational stability and solubility assay (Mean molar $[GdnHCl]_{1/2}$)	1.40 M	1.54 M	1.71 M *	1.38 M *

* Significant differences; NA: not applicable.

SUPPLEMENTARY FIGURE LEGENDS

Supplementary Figure S1. Western blot profiles and glycoform ratios of PK-treated and untreated PrP^{Sc} as well as presumed PrP^{C} from thalamus of sCJDMM2 and sFI. BH from thalamus of sCJDMM2 (N=3) and sFI (N=3) were processed as described in Figure 1. Analyses of totPrP^{Sc} (**A**), resPrP^{Sc} (**B**) and "PrP^{C}" (**C**) reveal that the differences in glycoform ratios between sCJDMM2 and sFI (**D-F**) are comparable to those observed in the frontal cortex (Fig. 1D, 1E and 1F): in thalamus preparations, as in frontal cortex, the glycoform ratios of totPrP^{Sc} significantly differ in the two diseases (**D**) while those of resPrP^{Sc} (**E**) and "PrP^{C}" (**F**) do not. However, in both diseases, "PrP^{C}" ratios demonstrated in the thalamus (**F)** differ from those of the frontal cortex (Fig. 1F). In **A-C**, a dashed line separates samples run on the same gel but not in adjacent lanes; in (**B**), a non-specific band of ~32 kDa band is also observed (arrow). Labels on the right side of each WB indicate the three PrP glycoforms: *D*: Di-glycosylated; *M*: Mono-glycosylated; *U*: Un-glycosylated.

Supplementary Figure S2. Characterization of the ~53 kDa and ~90 kDa components in sCJDMM2 and sFI. A-E: TotPrP^{Sc} from sCJDMM2 (odd number lanes) and sFI (even number lanes) were immunoprecipitated with antibody (Ab) 8B4 and then eluted either in sample buffer (samples 1, 2) or in urea-based buffer (samples 3, 4). A panel of Abs and various conditions were used for probing. **A**: 3F4; **B**: Only anti-mouse secondary Ab; **C**: Polyclonal Ab anti-N to PrP N-terminus; **D**: Polyclonal Ab 2301 to PrP C-terminus; **E**: Only anti-rabbit secondary Ab. **A**, **B**: Co-eluting Ab (arrows) is detected after elution in sample buffer, but not in urea-eluted samples. **A**, **C**, **D**: When Ab co-elution is abrogated, significant signal remains in the same WB region in sCJDMM2 but not in sFI. **F**, **G**: Incubation with PNGase F (**F**) and PK treatment (**G**) indicate that h.m.w. components are glycosylated and largely PK-sensitive. **H**, **I**: TotPrP^{Sc} obtained by

NaPTA precipitation of brain homogenates from sCJDMM2 and sFI was Western blotted and the relative quantity of h.m.w. components was estimated. The PrP^{Sc} contained in the R1-labelled ~45-70 kDa gel region (including the ~53 kDa component, overexposed) was expressed as ratio to PrP^{Sc} populating the region ~45-27 kDa (R2, including the traditional $totPrP^{Sc}$). High m.w. components are 3 to 5 times better represented in sCJDMM2 than in sFI but the difference is not as striking as in 2-D eluates (Fig. 3B) possibly because the h.m.w. components are concentrated during IP. Abs 3F4 and 8B4.

Supplementary Figure S3. $TotPrP^{Sc}$ glycoform ratios after sedimentation equilibrium. $TotPrP^{Sc}$ from sCJDMM2 and sFI cases were centrifuged at high speed for 19 hours in a 10-60% sucrose gradient. Identical aliquots were collected from each fraction and processed for WB (**A**). **B**: Quantitative analyses of the $totPrP^{Sc}$ glycoforms was carried out in the low-density (fractions 1-7 for sCJDMM2 and 3-9 for sFI) and high-density peaks (fractions 16-20). In sFI, the $totPrP^{Sc}$ glycoform ratio in the low-density fractions differs from that in high-density fractions ($P < 0.05$ for di- and un-glycosylated conformers) while in sCJDMM2 the glycoform ratio does not significantly change along the gradient. As a result of this divergence, glycoform ratios differ between sFI and sCJDMM2 in the low-density fractions but not in the high-density fractions. In sCJDMM2, glycoform ratios are 20:48:32 in peak 1-7 and 13:46:41 in peak 16-20; in sFI they are 41:50:9 in peak 3-9 and 24:45:31 in peak 16-20.

Supplementary Figure S4. Immunoprecipitation of sedimentation equilibrium fractions with the conformational antibody OCD4 that selectively immunoreacts with misfolded PrP. **A**: Relatively large quantities of PrP, likely $totPrP^{Sc}$, are recovered from sCJDMM2 and sFI fractions 4-8 (low-density) and 17-21 (high-density) but not from S1 and S2 fractions of a negative case, harboring mostly PrP^{C}. Note the weak signal in the S1 sample, likely representing

insoluble PrP[11]. **B**: Shorter exposure demonstrates the presence of the h.m.w components (arrows) in both sets of sedimentation fractions from sCJDMM2, but only in the 17-21 fractions from sFI, as well as the prominence of the di-glycosylated form in fractions 4-8 from sFI (arrowhead).

Supplementary Figure S5. Glycoform ratios of resPrP^{Sc} following sedimentation equilibrium centrifugation. Individual fractions of totPrP^{Sc} obtained from sCJDMM2 and sFI were digested with PK (10 U/ml) and processed for WB (**A**). A non-specific band of ~32 kDa is also observed (double arrow). **B**: The resPrP^{Sc} glycoform ratios in fractions 16-20, although apparently similar in profile, differ in their relative amounts of mono- and un-glycosylated forms (28:58:14 for sCJDMM2 and 28:43:29 for sFI).

Supplementary Figure S6. Sedimentation equilibrium of resPrP^{Sc} generated by PK treatment of totPrP^{Sc} from sCJDMM2 and sFI before fractionation. A: Sedimentation profile of resPrP^{Sc} demonstrating two partially overlapping but distinct peaks that populate fractions of lower density in sFI than in sCJDMM2 (8-18 in sFI vs. 14-21 in sCJDMM2). **B**: Quantitative analysis of glycoform ratios in the peaks of sCJDMM2 and sFI shows similar profiles in both diseases that also resemble those of previously unfractionated resPrP^{Sc}.

Supplementary Figure S1

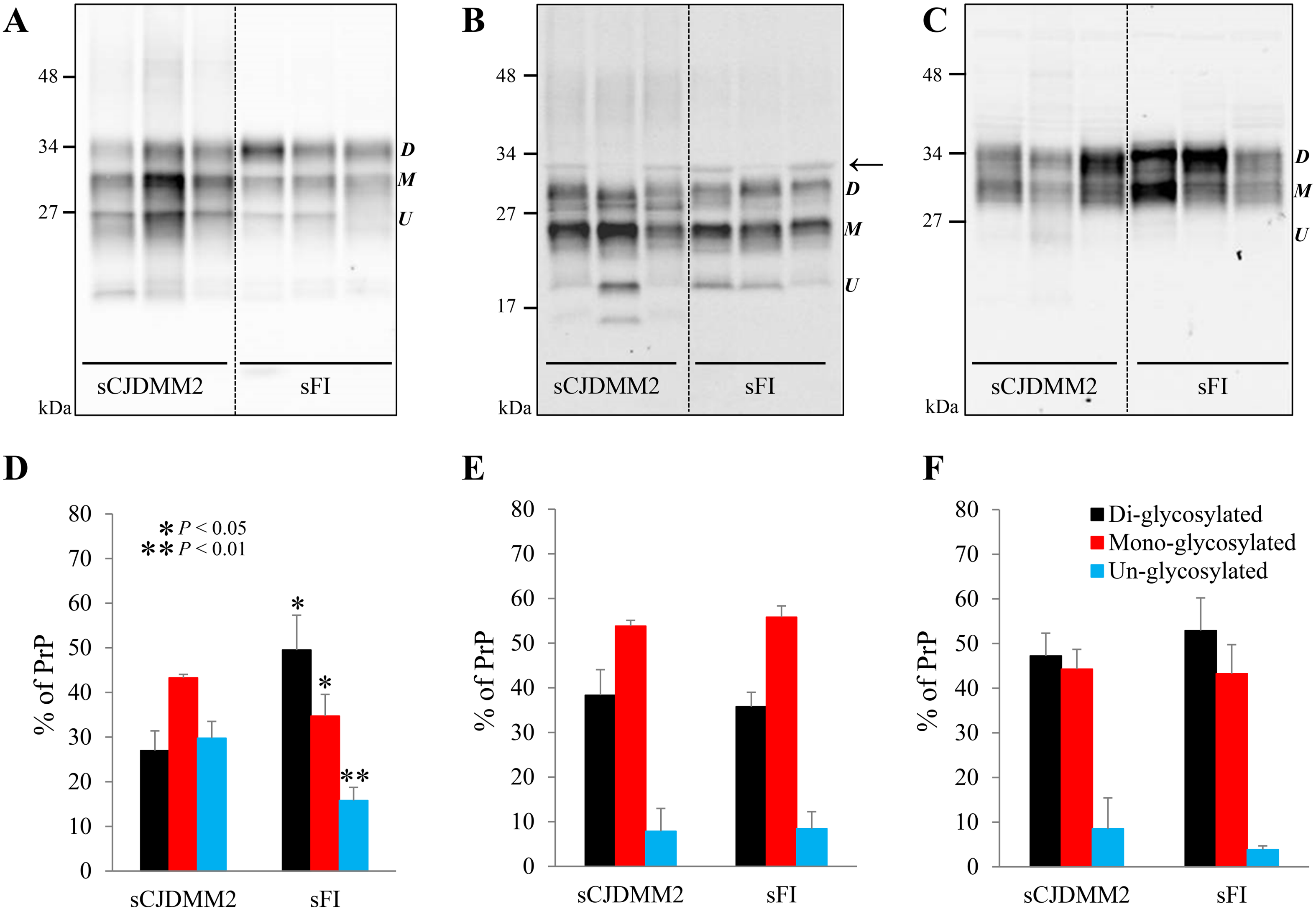

Supplementary Figure S2

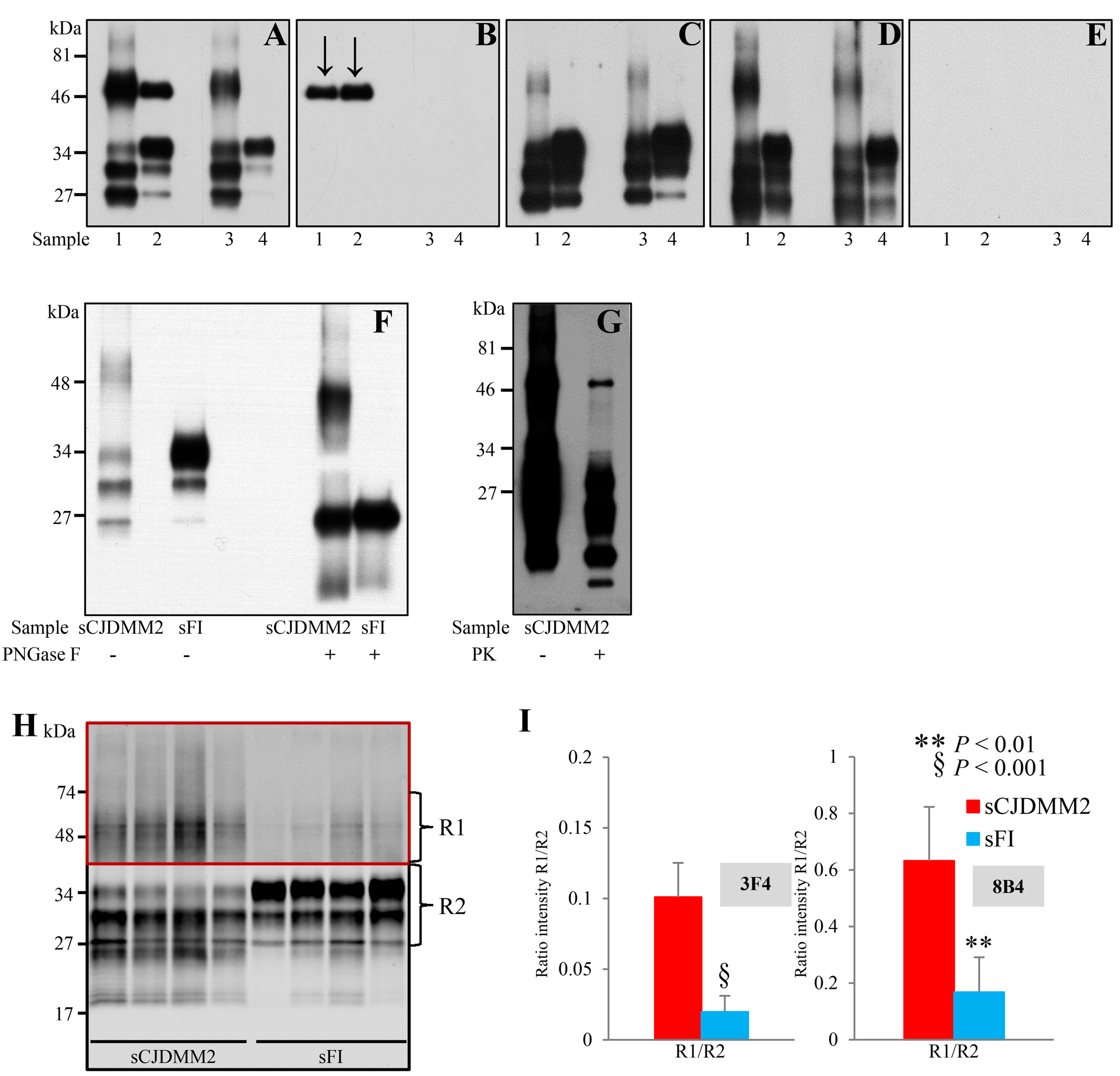

Supplementary Figure S3

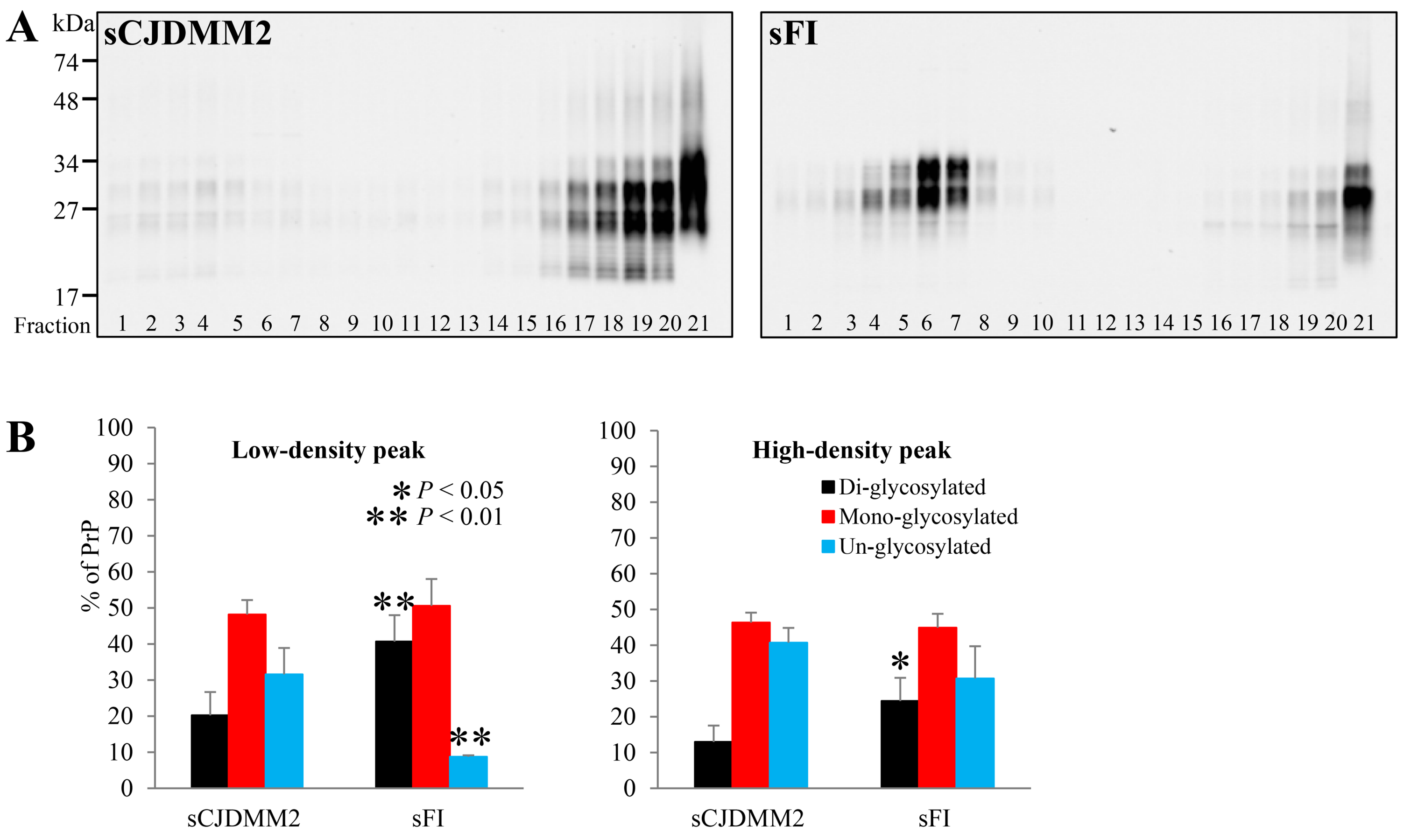

Supplementary Figure S4

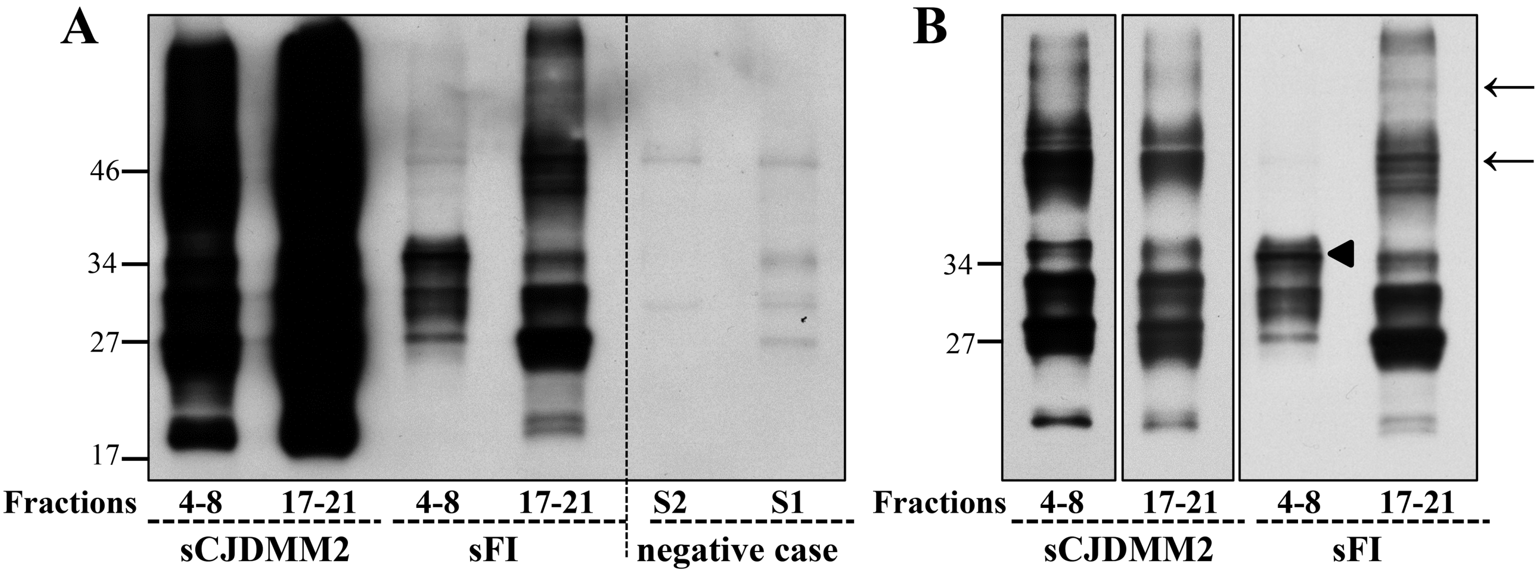

Supplementary Figure S5

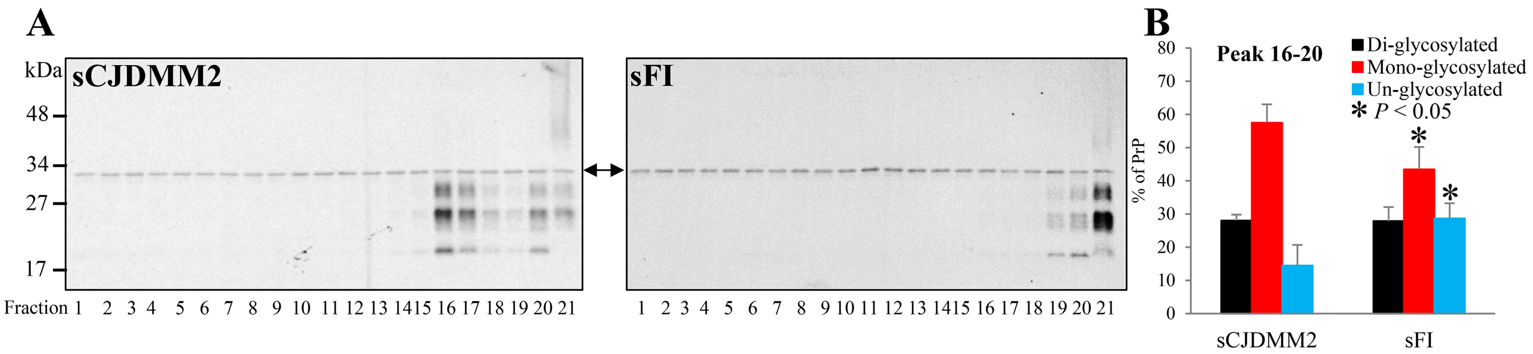

Supplementary Figure S6

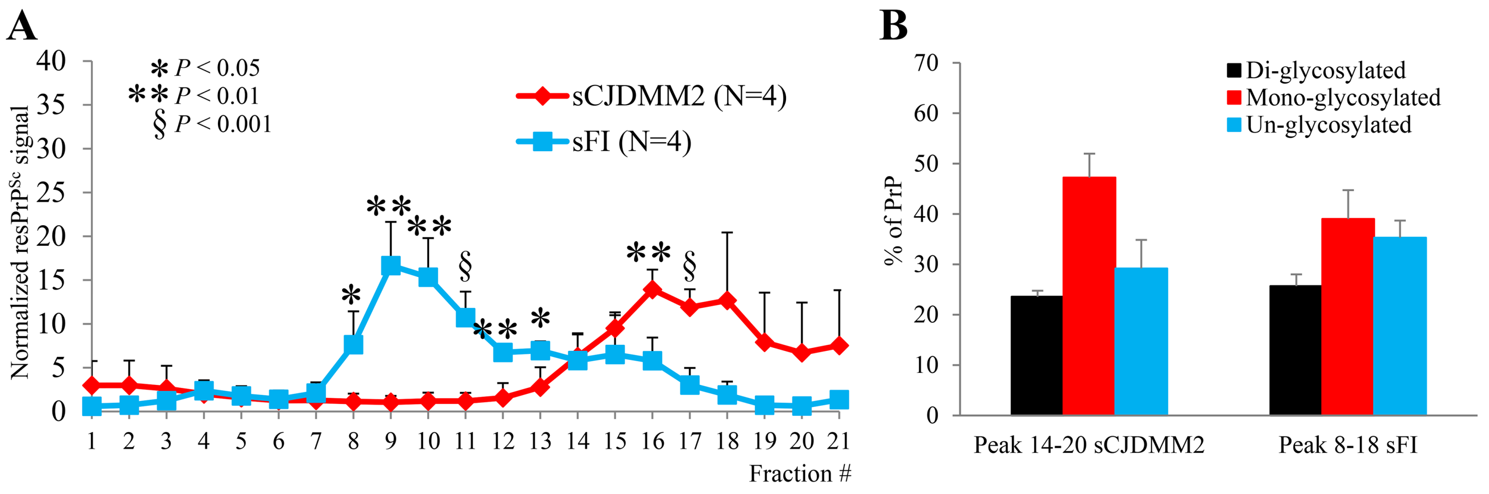

Supplement: Supplementary Information [file srep38280-s1.pdf]
